# Emotional Exhaustion of Burnout Among Medical Staff and Its Association With Mindfulness and Social Support: A Single Center Study During the COVID-19 Pandemic in Japan

**DOI:** 10.3389/fpsyt.2022.774919

**Published:** 2022-03-15

**Authors:** Makiko Sampei, Ryo Okubo, Mitsuhiro Sado, Aurelie Piedvache, Tetsuya Mizoue, Koushi Yamaguchi, Naho Morisaki

**Affiliations:** ^1^Department of Health Science, Health Promotion, Nippon Sport Science University, Tokyo, Japan; ^2^Department of Social Medicine, National Research Institute for Child Health and Development, Tokyo, Japan; ^3^Translational Medical Center, National Center of Neurology and Psychiatry, Tokyo, Japan; ^4^Department of Neuropsychiatry, Keio University School of Medicine, Tokyo, Japan; ^5^Department of Epidemiology and Prevention, Center for Clinical Sciences, National Center for Global Health and Medicine, Tokyo, Japan; ^6^Center of Maternal-Fetal, Neonatal and Reproductive Medicine, National Center for Child Health and Development, Tokyo, Japan

**Keywords:** burnout, social support, mindfulness, healthcare worker, emotional exhaustion

## Abstract

**Background:**

Although higher rates of burnout have been reported during the COVID-19 pandemic, the contribution of the modifiable factors is lesser-known. We investigated how the risk of emotional exhaustion was associated with mindfulness skills and social support in a single medical center in Japan.

**Methods:**

We conducted a cross-sectional web survey on mental health for all staff of a national medical hospital from February to March 2021. We examined the association between self-rated emotional exhaustion and levels of mindfulness and social support using multivariate logistic regression.

**Results:**

Of the 830 participants, signs of emotional exhaustion were observed in 261 (31%) individuals. Among those highly exposed to the virus at work, individuals with low levels of mindfulness and social support had significantly higher odds of emotional exhaustion [OR 3.46 (95% CI; 1.48–8.09), OR; 3.08 (95% CI; 1.33–7.13), respectively] compared to those with high levels. However, among those not highly exposed to the virus, individuals with both low and moderate levels of mindfulness had significantly higher odds of emotional exhaustion. [OR 3.33 (95% CI; 2.22–5.00), OR; 2.61 (95% CI; 1.73–3.94), respectively].

**Conclusion:**

We found that factors associated with emotional exhaustion differed by exposure to SARS-CoV-2. Building mindfulness skills can help reduce the high burden placed on the staff. Additionally, increasing social support may be useful especially for workers highly exposed to SARS-CoV-2.

## Introduction

The current COVID-19 worldwide pandemic has continued to impose a tremendous burden on healthcare workers (1, 2), whether or not they treat COVID-19 patients directly (3–5). Previous studies have suggested that possibly one-third of the healthcare workers may have reached the point of emotional exhaustion or burnout (5, 6). Burnout is marked by any or all of the following characteristics: energy depletion or exhaustion, depersonalization or increased mental distance from one's job, reduced professional efficacy, or a decreased sense of personal accomplishment (7, 8). During a pandemic, sustaining the healthcare system is vital. Thus, identifying factors and measures that reduce the risk of burnout and emotional exhaustion among healthcare workers should be critical.

In the midst of the pandemic in which the rapid response to the environment, which continues to change drastically, is required, it would be difficult to sufficiently modify a work environment in order to reduce the major stressors for healthcare professionals, which have been reported to be causes of emotional exhaustion: increased workload, personal risk of infection, fear of transmission to family members, illness or death of friends and colleagues, and loss of many patients (3, 9–11). In addition, it is also impossible to alter personal characteristics, such as ethnicity and pre-existing psychiatric history, even though studies have shown these may influence one's risk of experiencing emotional exhaustion. Therefore, identifying factors modifiable through education or skill-building which could prevent emotional exhaustion is more reasonable. Previous studies indicate that some factors: mindfulness (12–14), social support (10, 15–17), may ameliorate the risk of emotional exhaustion and mental health.

Mindfulness is a mental state defined as “paying of purposeful non-judgmental attention to the present moment (18–20). Knowledge of the previous intervention studies which showed that mindfulness-based interventions are effective for reducing the risk of emotional exhaustion (12–14) implies that the mental state of mindfulness is linked to the risk of burnout. However, we should be aware that caution is raised for these findings in terms of the quality of the previous studies, etc., (21). Therefore, the relationship between mindfulness and the risk of emotional exhaustion in the midst of pandemics is still unclear. Social support is defined as “the provision of assistance or comfort to others, typically to help them cope with biological, psychological, and social stressors” (22). Several studies have shown that social support may reduce stress and the risk of emotional exhaustion among nurses under the COVID-19 pandemic; however, such studies have not been conducted among other professionals (10, 17, 23, 24).

In addition, effective interventions may differ among workers who whether or not have experience of high-exposure work to SARS-CoV-2. However, we could not identify any studies that focused on the same.

Thus, in this study, we investigated the association between the risk of emotional exhaustion and mindfulness and social support among workers with and without high-exposure work to SARS-CoV-2 in a single medical center in Japan.

## Materials and Methods

### Participants and Procedure

In this cross-sectional study, we sent an email to all staff members of the National Research Institute for Child Health and Development, including the medical doctors, nurses, other workers such as medical technicians, and administrative or management staff, and invited them to participate. Clicking on the URL in the email opened the web questionnaire response page, where participants could answer the questions. We sent reminder e-mails three times: one in early March, one in 2 weeks before the end of the application period, and one 3 days before the end of the application period. Participants who consented to the study were asked to complete an online questionnaire using “Microsoft Forms,” an online software for surveys provided by Microsoft. The survey questions included those pertaining to emotional exhaustion, mindfulness, social support, and high-exposure work to SARS-CoV-2. If the participant also consented and allowed the use of their personal information, which was otherwise anonymized in the research ID from the hospital administrative department, we obtained demographic data such as age and gender. The reason behind getting these data is to reduce the burden on the participants (medical staff during the pandemic) by answering fewer questions on the questionnaire. The National Research Institute for Child Health and Development has 490 hospital beds and a research center. It is one of the national centers in Japan and is located in the western part of Tokyo. It usually provides specialized treatment for all diseases in children. Due to increasing COVID-19 infected patients, the center started accepting COVID-19 infected inpatients from October 2020. The survey was conducted from February 15th to March 19th, 2021. This period was during the third wave of the pandemic in Japan, and the number of infected people increased daily.

### Exclusion and Inclusion Criteria

Inclusion criteria were that (1) they are a staff member who works at the National Research Institute for Child Health and Development, either paid or unpaid. (2) they have a unique email address given by the institution.

Exclusion criteria were not willing to participate in our study, and those with missing responses for emotional exhaustion, mindfulness, and social support responded were excluded.

## Measurements

### Emotional Exhaustion

Emotional exhaustion was measured using the single-item measure of burnout (SMB), “Overall, based on your definition of burnout, how would you rate your level of burnout?” (25) The SMB's possible responses were (1) “I enjoy my work. I have no symptoms of burnout,” (2) “Occasionally I am under stress, and I do not always have as much energy as I once did, but I do not feel burned out,” (3) “I am definitely burning out and have one or more symptoms of burnout, such as physical and emotional exhaustion,” (4) “The symptoms of burnout that I'm experiencing won't go away. I think about frustration at work a lot” or (5) “I feel completely burned out and often wonder if I can go on. I am at the point where I may need some changes or may need to seek some sort of help.”

The SMB was generated by choosing the one-item of the Mini-z (26–28), a questionnaire that measured burnout among healthcare workers, developed by the American College of Physicians (ACP). The Japanese version was created and validated by the ACP Japan group (29). We used the single-item version which Rohland et al. (25) validated against the Maslach Burnout Inventory (MBI), which has three subscales as emotional exhaustion, personal accomplishment, and depersonalization, which is currently understood to be the gold standard burnout measurement (30, 31). Rohland reports the single-item to be correlated at *r* = 0.64 (*p* < 0.0001) with emotional exhaustion and the ANOVA yielded an R2 of 0.5 (*p* < 0.0001). Thus, in this paper, we capture the emotional exhaustion of burnout.

We followed the definition of previous studies (26, 32) and defined burnout for the descriptive report by answering either (3), (4) or (5), (3) “I am definitely burning out and have one or more symptoms of burnout, such as physical and emotional exhaustion”; (4) “The symptoms of burnout that I'm experiencing won't go away. I think about frustration at work a lot”; or (5) “I feel completely burned out and often wonder if I can go on. I am at the point where I may need some changes or may need to seek some sort of help.” We treated the burnout scale as an ordinal variable and used multiple ordered logistic regression in the main analysis.

### Mindfulness

Mindfulness, defined as “the awareness of one's internal states and surroundings,” is a concept that has been applied to various therapeutic interventions. These include mindfulness-based cognitive behavior therapy, mindfulness-based stress reduction, and mindfulness meditation that help people avoid “the destructive or automatic habits and responses by learning to observe their thoughts, emotions, and other present-moment experiences without judging or reacting to them” (33).

The Mindful Attention Awareness Scale (MAAS) (34), a unidimensional scale, was used. The scale comprised 15 items, with each item rated on a 6-point Likert scale, ranging from 1 (almost always) to 6 (almost never), and measured mindfulness in everyday experience. Some examples were, “I could be experiencing some emotion and not be conscious of it until sometime later” and “I tend not to notice feelings of physical tension or discomfort until they really grab my attention.” The total scores ranged from 15 to 90, and a higher score reflected a higher level of mindfulness. We used the Japanese version of the MAAS (35). For this analysis, the variable was transformed into three categories based on terciles of the total score distribution.

### Social Support

Social support is defined as “the provision of assistance or comfort to others, typically to help them cope with biological, psychological, and social stressors. Support may arise from any interpersonal relationship in an individual's social network, involving family members, friends, neighbors, religious institutions, colleagues, caregivers, or support groups. It may take the form of practical help (e.g., doing chores, offering advice), tangible support that involves giving money or other direct material assistance, and emotional support that allows the individual to feel valued, accepted, and understood” (22).

The Multidimensional Scale of Perceived Social Support (MSPSS) (36), developed and validated by Zimet et al., consists of 12 items rated on a 7-point Likert scale (1 = very strongly disagree; 7 = strongly agree), designed to measure perceived social support from three-domain for family, friends, and significant other. The Japanese 7-item version that chose seven of the original 12 items was translated and validated by Iwasa et al. (37). The seven items were: “There is a special person who is around when I am in need”; “There is a special person with whom I can share my joys and sorrows”; “My family really tries to help me”; “I get the emotional help and support I need from my family”; “My friends really try to help me”; “I have friends with whom I can share my joys and sorrows”; or “I can talk about my problems with my friends.” The total scores ranged from 7 to 49, and higher scores implied a greater level of perceived social support. For this analysis, the variable was transformed into three categories based on terciles of the total score distribution.

### Potential Exposure to the SARS-CoV-2

We asked participants if they conducted specific tasks at work that would potentially expose them to SARS-CoV-2 (response items were yes/no). Such specific tasks were defined as the following: “Intubation and extubation of respirators for COVID-19 patients, and/or worked in close proximity to them,” “Collected specimens from the COVID-19 patients from the nasal cavity and pharynx, and/or worked in close proximity to them,” “Performed operations on patients and/or worked in close proximity to them,” and “Other work in spaces where there was a possibility of high levels of SARS-CoV-2.” Those who found any of the above list applicable were to select “Yes”. In this study, we defined participants who responded “yes” as “the highly exposed” group, and “no” as “the not highly exposed” group.

### Sociodemographic and Other Characteristics

The participants self-reported their demographic characteristics such as education, job type, years of current work, marital status, and whether they had children. Data on age and sex were retrieved from the hospital administrative data. For analysis, we categorized age and years of current work, referring to previous studies (3, 38, 39).

### Statistical Analysis

We reported, for descriptive purposes, the means and standard deviations for age and years of current work but used them as a category in the main analysis. Additionally, we calculated the proportions for the categorical variables for all samples and each emotional exhaustion group and no emotional exhaustion group. We assessed the difference between these two groups with a chi-square test. We conducted a test using the Benjamini–Hochberg method to reduce the risk of making Type I error. Cronbach's alpha coefficients were calculated to test internal validity for the Mindful Attention Awareness Scale (MAAS) and Multidimensional Scale of Perceived Social Support (MSPSS) (see Supplementary Document).

Next, we examined the distribution of the participants' emotional exhaustion responses and examined the association between emotional exhaustion and mindfulness and social support using a multiple ordered logistic regression model. It was conducted overall and stratified by whether or not the participant had high exposure to SARS-CoV-2. Also, we conducted the analysis stratified by job type. Since we used the emotional exhaustion scale that only included one item, we conducted a sensitivity analysis that we performed ordered logistic regression using PHQ-9 (40, 41), a conceptual measure of depression, as the outcome. Based on a previous study (42), we treated it as an ordered variable by severity and showed the distribution of participants who responded to PHQ-9 (Supplementary Table 3). All analyses were conducted using STATA/MP 17.0 software (Stata Corp Drive, College Station, TX, USA). *P*-values <0.05 were defined as statistically significant.

### Ethical Considerations

We obtained informed consent from all respondents in our study. Our study was approved by the ethics committee of the National Research Institute for Child Health and Development (2020-266). The study was conducted following- the code of ethics set by the Declaration of Helsinki and all its future amendments or comparable standards.

## Results

### Characteristics of the Participants and Emotional Exhaustion Prevalence, Distributions

**Figure 1:**
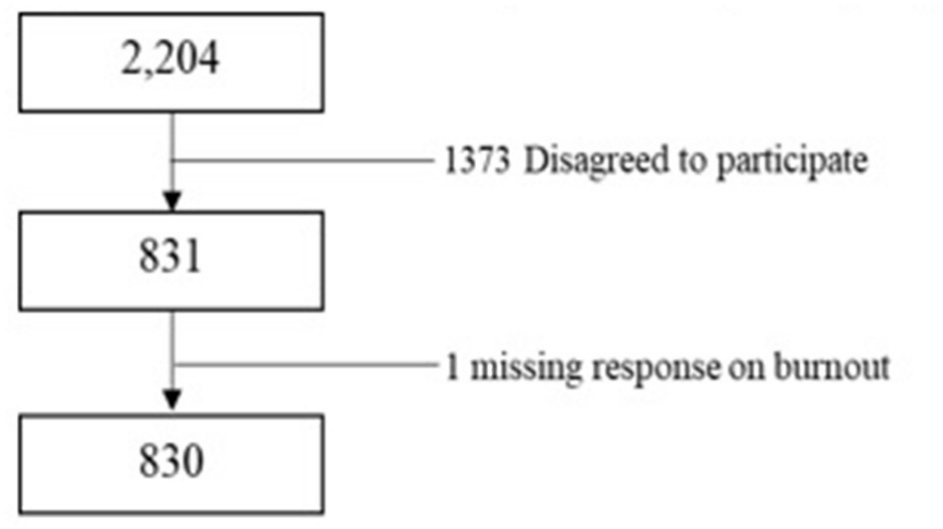
Flowchart of analysis data for participants.

Out of the 2,204 people who received the study invitation email, 831 (38%) completed the survey. One person having missing information on emotional exhaustion was excluded (Figure 1). Thus, 830 samples were included in our analysis. The participants were aged 41 on average (Standard Deviation; SD; 11), and had worked at the institute for around 4.7 years (SD; 3.7). Most respondents were females (74.7%), and the occupation distribution was as follows: 23.7 % medical doctors, 34.0% nurses, 13.1% other medical staff, and 29.2% office workers or researchers (Table 1).

**Table 1:** Demographic characteristics all of the participants and by emotional exhaustion.

		**Total**	**Emotional exhaustion**		
			**No (*N* = 569)**	**Yes (*N* = 261)**	***P***
		***N* (%)**	***N* (%)**	***N* (%)**	
Age (years)	22–35	306 (36.9)	207 (68)	99 (32)	0.13
	36–50	346 (41.7)	229 (66)	117 (34)	
	>51	178 (21.5)	133 (75)	45 (25)	
Years of current work^*^	<3 years	372 (44.8)	270 (73)	102 (27)	<0.01
	≥3 years	446 (53.7)	287 (64)	159 (36)	
	Missing	12 (1.5)	12 (100)	0 (0)	
Sex	Male	210 (25.3)	147 (70)	63 (30)	0.60
	Female	620 (74.7)	422 (68)	198 (32)	
Job type^*^	Doctor	197 (23.7)	151 (77)	46 (23)	<0.01
	Nurse	282 (34.0)	166 (59)	116 (41)	
	Other medical staff	109 (13.1)	79 (72)	30 (28)	
	Office worker or researcher	242 (29.2)	173 (71)	69 (29)	
High dose exposure to SARS-CoV-2	No	653 (78.7)	455 (70)	198 (30)	0.18
	Yes	177 (21.3)	114 (64)	63 (36)	
Married^*^	No	344 (41.5)	217 (63)	127 (37)	<0.01
	Yes	486 (58.6)	352 (72)	134 (28)	
Have children	No	518 (62.4)	352 (68)	166 (32)	0.63
	Yes	312 (37.6)	217 (70)	95 (30)	
Mindfulness^*^	Lower	292 (35.2)	169 (58)	123 (42)	<0.01
	Moderate	267 (32.2)	175 (66)	92 (34)	
	Higher	271 (32.7)	225 (83)	46 (17)	
Social support^*^	Lower	278 (33.5)	160 (58)	118 (42)	<0.01
	Moderate	294 (35.4)	215 (73)	79 (27)	
	Higher	258 (31.1)	194 (75)	64 (25)	

*N, number %, proportion; p, chi-squared test p-value*.

* *Chi-squared test p < 0.05*.

Among the participants, 21.3% reported being highly exposed to SARS-CoV-2. The overall prevalence of emotional exhaustion was 31%, with 36% and 30% in the highly exposed and not highly exposed groups, respectively. By profession, nurses (41%) had the highest prevalence of burnout compared to doctors (23%), other medical staff (28%), and office workers (29%) (*p* < 0.05).

Participants who had no symptoms of emotional exhaustion, defined as those who selected “No symptoms” and “Do not feel burned out” was 69% of the study population. Participants who had signs of emotional exhaustion, defined as those who selected “Have one or more symptoms of emotional exhaustion,” “Have symptoms always,” and “Feel completely burned out” were 31% of the study population (Table 2).

**Table 2:** Distribution of the participants' emotional exhaustion responded.

	**Frequency**	**Percent**
No symptoms^a^	73	8.8
Do not feel burned out^b^	496	59.8
Have one or more symptoms of burnout^c^	158	19.0
Have symptoms always^d^	94	11.3
Feel completely burned out^e^	9	1.1
Total	830	100

a *“I enjoy my work. I have no symptoms of burnout”*.

b *“Occasionally I am under stress, and I do not always have as much energy as I once did, but I do not feel burned out”*.

c *“I am definitely burning out and have one or more symptoms of burnout, such as physical and emotional exhaustion”*.

d *“The symptoms of burnout that I'm experiencing won't go away. I think about frustration at work a lot”*.

e *“I feel completely burned out and often wonder if I can go on. I am at the point where I may need some changes or may need to seek some sort of help”*.

### Relationship Between Emotional Exhaustion and Modifiable Factors

Table 3 shows the association between the odds of burnout and the degree of mindfulness and social support using multiple ordered logistic regression analyses.

**Table 3:** The association between odds of emotional exhaustion and the degree of mindfulness and social support among the participants and by exposure to SARS-CoV-2 using ordered logistic regression.

		**All samples**			**The virus-exposed**			**The no-exposure**		
		**OR**	**95%CI**	***p* for trend**	**OR**	**95%CI**	***p* for trend**	**OR**	**95%CI**	***p* for trend**
Mindfulness	Lower	3.39	2.36–4.87	<0.01	3.46	1.48–8.09	<0.01	3.33	2.22–5.00	<0.01
	Moderate	2.20	1.53–3.17		1.47	0.62–3.44		2.61	1.73–3.94	
	Higher	Reference			Reference			Reference		
Social support	Lower	1.89	1.31–2.73	<0.01	3.08	1.33–7.13	0.02	1.65	1.09–2.50	0.01
	Moderate	1.27	0.89–1.80		1.70	0.79–3.69		1.12	0.75–1.68	
	Higher	Reference			Reference			Reference		

*Adjusted age, sex, years of current work, job type, married, have children, High dose exposure to SARS-CoV-2 (for only all samples)*.

*OR, odds ratio; 95%CI, 95% confidence interval*.

In the total sample, we observed significant associations between burnout and tertiles of mindfulness and tertiles of social support in the univariate ordered logistic regression. We judged that the results we got were not coincidental because the test results using the Benjamini–Hochberg show that all *p*-value is significant even after multiple adjustments.

In the adjusted analysis (Table 3), compared to those with a higher level of mindfulness, moderate and lower levels of mindfulness had significantly higher odds of emotional exhaustion [Odds ratio; OR; 2.20 95% CI: Confidential Interval; 1.53–3.17), OR; 3.39 (95% CI; 2.36–4.87)], respectively. Compared to those with a higher level of social support, those with lower levels had significantly higher odds of emotional exhaustion [OR; 1.89 (95% CI; 1.31–2.73)]; however, the odds were not significantly different for those with a moderate level of social support [OR; 1.27 (95% CI; 0.89–1.80)]. Coefficients for all variables included in the model are shown in the Supplementary Table 1. The results of the relationship between depression and modifiable factors for sensitivity analysis were similar. Moderate and lower levels of mindfulness had significantly higher odds of depression compared to those with a higher level of mindfulness. Compared to those with a higher level of social support, those with lower levels had significantly higher odds of depression (Supplementary Table 3).

### Relationship Between Emotional Exhaustion and Modifiable Factors, Stratified by Exposure to SARS-CoV-2

The association between the risk of burnout and the degree of mindfulness and social support stratified by exposure to SARS-CoV-2 is shown in Table 3. Among those highly exposed, participants with a lower level of mindfulness, compared to those with a higher level, had significantly higher odds of emotional exhaustion [OR; 3.46 (95% CI; 1.48–8.09)]; however, the risk was not significantly different for those with a moderate level [OR; 1.47 (95% CI; 0.62–3.44)]. Compared to those with a higher level of social support, those with a lower level had significantly higher odds of emotional exhaustion [OR; 3.08 (95% CI; 1.33–7.13)]; however, the odds were not significantly different for those with a moderate level [OR; 1.70 (95% CI; 0.79–3.69)]. P for trend was significant for both mindfulness and social support levels.

In the not highly exposed group, both the moderate and low mindfulness groups had significantly higher odds of burnout [OR; 2.61 (95% CI; 1.73–3.94), OR; 3.33 (95% CI; 2.22–5.00), respectively] compared to those with a higher level of mindfulness.

Compared to those with a higher level of social support, those with a lower level had significantly higher odds of emotional exhaustion [OR; 1.65 (95% CI; 1.09–2.50)]; on the other hand, the odds were not significantly different for those with a moderate level [OR; 1.12 (95% CI; 0.75–1.68)]. We observed a significant trend (*p*-value < 0.01).

### Relationship Between Emotional Exhaustion and Modifiable Factors, Stratified by Job Type

The association between the risk of emotional exhaustion and the degree of mindfulness and social support stratified by job type is shown in Table 4.

**Table 4:** The association between odds of emotional exhaustion and the degree of mindfulness and social support among the participants and by job type using ordered logistic regression.

		**Medical doctor**			**Nurse**			**Other medical staff**			**Office worker or researcher**		
		**OR**	**95%CI**	***p* for trend**	**OR**	**95%CI**	***p* for trend**	**OR**	**95%CI**	***p* for trend**	**OR**	**95%CI**	***p* for trend**
Mindfulness	Lower	4.30	1.93–9.59	<0.01	2.89	1.55–5.37	<0.01	5.27	1.76–15.80	<0.01	2.55	1.29–5.05	<0.01
	Moderate	3.17	1.42–7.06		2.19	1.17–4.08		2.21	0.72–6.82		2.12	1.07−4.18	
	Higher	Reference			Reference			Reference			Reference		
Social support	Lower	4.07	1.65–10.02	<0.01	1.32	0.71–2.46	0.73	0.83	0.27–2.56	0.48	2.33	1.19–4.55	0.02
	Moderate	2.36	1.04–5.34		1.02	0.58–1.81		0.65	0.22–1.91		1.25	0.64–2.45	
	Higher	Reference			Reference			Reference			Reference		

*Adjusted age, sex, years of current work, married, have children, high dose exposure to SARS-CoV-2*.

*OR, odds ratio; 95% CI, 95% confidence interval*.

Among medical doctors, emotional exhaustion risk was significantly higher in both the low and moderate groups than in the high group for both mindfulness [Odds ratio; OR; 4.30, 95% CI: Confidential Interval; 1.93–9.59), OR; 3.17 (95% CI; 1.42–7.06)] and social support [OR; 4.07, 95% CI; 1.65–10.02), OR; 2.36 (95% CI; 1.04–5.34)], respectively. These dose-response relationships measured by *p*-value for trend were also significant. Among nurses, burnout risk was significantly higher in both the low and moderate groups compared to the high group for mindfulness [OR; 2.89, 95% CI; 1.55- 5.37), OR; 2.19 (95% CI; 1.17–4.08)], however, the increase in risk was not significant for social support [OR; 1.32, 95% CI; 0.71–2.46), OR; 1.02 (95% CI; 0.58–1.81)], and we failed to observe a significant dose-response relationship.

Among other medical staff, there was a significant higher risk of burnout in the low mindfulness group compared to the high mindfulness group [OR; 5.27 (95% CI; 1.76–15.80)], but no significant increase in risk was observed in the moderate group [OR; 2.21 (95% CI; 0.72–6.82)]. Similar to nurses, we failed to observe a significant relationship between social support and emotional exhaustion [OR; 0.83, 95% CI; 0.27–2.56), OR; 0.65 (95% CI; 0.22–1.91)], or a significant dose-response relationship.

Among administrative and research staff, emotional exhaustion risk was significantly higher in both the low and moderate groups compared to the high mindfulness group [OR; 2.55, 95% CI; 1.29–5.05), OR; 2.12 (95% CI; 1.07–4.18)]. There was a significant relationship with emotional exhaustion in the low social support group compared to the high social support group [OR; 2.33 (95% CI; 1.19–4.55)], but no significant relationship was observed in the moderate group [OR; 1.25 (95% CI; 0.64–2.45)].

## Discussion

In this one-hospital study, we found that the factors associated with emotional exhaustion differed by whether the worker had high exposure to SARS-CoV-2 and if medical doctor. Lower levels of mindfulness were associated with higher odds of emotional exhaustion regardless of exposure; however, lower levels of social support were significant odds only among those with high exposure to SARS-CoV-2. Lower levels of social support were associated with higher odds of emotional exhaustion among only medical doctors.

The overall prevalence of emotional exhaustion was 31% in our study. A meta-analysis on emotional exhaustion among healthcare workers during the COVID-19 pandemic reported that the prevalence was 34.4% (2), similar to our study, and 31.4 %, as reported at the early stages of the pandemic in Japan (5). It is noteworthy that in our research and previous Japanese studies (5), nurses had the highest prevalence compared to doctors and other professionals. In non-pandemic settings, a meta-analysis estimated that burnout among nurses (11%) was low compared to those reported from medical and surgical residents (15.4–51%) (43) and among emergency medicine physicians (35–40%) (44–47). This difference may be due to the specific situation of the pandemic, where the additional measures required, such as the use of unfamiliar personal protective equipment, application of zoning, and increased workload due to infection prevention work addition, altered the work environment of nurses the most.

In our study, social support and mindfulness, mindfulness was the sole factor that showed a significant association on the odds of emotional exhaustion in both groups. While our result is the first to indicate that high levels of mindfulness possibly have a protective effect on emotional exhaustion during the COVID-19 pandemic, the results were consistent with previous studies, showing the effectiveness of mindfulness interventions on mental health and emotional exhaustion in non-pandemic situations (14, 48).

High levels of mindfulness can improve metacognition, a decentered awareness mode where negative thoughts and feelings could be seen as passing events (49–51), helping externalize one's thoughts and emotions and observe one's status objectively. In a pandemic setting, where job and personal stress may increase, a health worker can be exhausted easily by endless contemplation about various difficulties, such as the patient's severe condition and the inability to provide usual care to their patients. Additionally, they anticipate the risk of infection, the possibilities of their infecting other patients or their family members, including their children, the risk of their children being discriminated against due to their job, or economic difficulties of oneself or family. Furthermore, the uncertainty of the convergence of the pandemic boosts distress, leading to enhanced rumination. Such ruminations can wear out a person and lead to emotional exhaustion (52, 53). By mindfully accepting experiences instead of perseverating on them, rumination decreases, and one is more likely to notice conditions (e.g., tiredness, exhaustion, etc.). Therefore, it becomes easier to include adaptive or healthier activities into our lives, such as adjusting the schedule, avoiding unnecessary information, and getting enough rest to evade ruminations (54). Such behavior change possibly prevents people from emotional exhaustion. Both reducing negativity and improving positivity, mindfulness skills have proved to improve psychological positivity (55). It could work as the protective factor for burnout for broadening the scope of attention to encompass pleasurable and meaningful events and thereby build motivation toward purposeful engagement in life (56). As mindfulness skills can be taught, our results suggest intervention benefits to personally enhance mindfulness among medical professionals not only in normal times but also during disasters, like the COVID-19 pandemic.

We also observed that higher social support was associated with reduced odds of emotional exhaustion only among the highly exposed group. Previous studies have suggested that social support is protective against emotional exhaustion among nurses working during the COVID-19 pandemic (10, 17) and non-pandemic situations (15, 16). While it is unclear whether our results differ from previous studies, several possibilities are explaining this disparity. One possibility is the difference in the population. Previous studies included only nurses, while in this study, the highly exposed group had more nurses than the not highly exposed group, which included administrative and management staff. Nurses, who were the target population of the previous studies, may have had more opportunities to work with support from team members than other healthcare professionals because they have more opportunities to work with patients in teams than other medical staff. Alternatively, “social distancing” required to prevent the spread of COVID-19 may have inhibited the protective effect of social support on maintaining the mental health of other professionals. Another possibility is reverse causality (57), that is, in our study, people who had more social support were those who possessed worse mental health and higher needs (58).

In our analysis stratified by job type, medical doctors with lower social support showed higher risk of emotional exhaustion compared to those with high social support, an association which was insignificant for other staff. The mechanism for this is unclear in this study, but may be related to differences in job characteristics between medical doctors and other staffs. Medical doctors play a greater role in explaining to and discussing about medical conditions and treatments options with patients and their families, and in deciding on which treatment to use compared to other staffs. Taking on such a role in an unprecedent infectious disease such as COVID-19, is likely a large burden for medical doctors. As higher social support may lead to easier information sharing consultation with peer medical doctors, it may have had a greater impact on reducing risk of emotional exhaustion among medical doctors compared to other staff. Further research in a multi-institutional setting is required to further investigate this hypothesis.

### Limitations and Strength

Our study has several limitations. Firstly, there was a possibility of sampling bias as only one hospital was included, with a participation rate of just 38% due to voluntary participation. Therefore, we admit that limitations might remain relevant to the external validity of the results obtained. We, however, compared percentages of each characteristic (e.g., age, sex, and job type) across the institute with those of the participants and found the two groups were very similar. Furthermore, the purpose of this study is to examine the association of mindfulness and social support with the risk of emotional exhaustion. Since the possible confounders for this association were adjusted for in the multivariate analysis, the low response rate was not considered fatal to the study's internal validity. Second, those at higher risk for emotional exhaustion may have been less likely to participate in our study. Hence, the results may have been underestimated, as the participants with high odds of burnout were possibly not included. Third, this study was a cross-sectional investigation and did not compare the results with those before the COVID-19 pandemic. Therefore, we do not know whether the associations are causal. Forth, the measurements in this study are by self-report similar to a previous mindfulness intervention study (21). As all questions were asked in one survey, we cannot exclude the possibility of common methods bias (59). However, detrimental bias was not detected in the correlation matrix procedure. A more appropriate approach would have been to implement the instrumental variable (IV) technique (59). However, we were unable to as we did not have a variable that would meet the requirements to be a strong IV to properly examine the association of mindfulness and social support with emotional exhaustion (59).

The strengths of our study are the relatively large sample size and the investigation of modifiable factors which could guide future interventions for emotional exhaustion in health care workers during the pandemic situation.

## Conclusion

We found that the factors associated with emotional exhaustion differed by exposure to COVID-19. Interventions building mindfulness seem promising in reducing the high burden placed on the staff; however, an increase in social support may also be beneficial, especially for workers exposed to high doses of the COVID-19 virus. Future research should consider longitudinal studies where data is collected by random sampling.

## Data Availability Statement

The raw data supporting the conclusions of this article will be made available by the authors, without undue reservation.

## Ethics Statement

The studies involving human participants were reviewed and approved by the Ethics Committee of the National Research Institute for Child Health and Development. Written informed consent for participation was not required for this study in accordance with the national legislation and the institutional requirements.

## Author Contributions

MS and NM initiated the idea. MS, RO, and NM designed the study. MS, NM, and KY performed the investigation. AP prepared the data. MS analyzed the data and wrote the draft of the study. All authors provided the input to the draft and contributed to the article and approved the submitted version.

## Funding

This work was supported by the Japan Health Research Promotion Bureau Research Fund (2020-B-09).

## Conflict of Interest

The authors declare that the research was conducted in the absence of any commercial or financial relationships that could be construed as a potential conflict of interest.

## Publisher's Note

All claims expressed in this article are solely those of the authors and do not necessarily represent those of their affiliated organizations, or those of the publisher, the editors and the reviewers. Any product that may be evaluated in this article, or claim that may be made by its manufacturer, is not guaranteed or endorsed by the publisher.
