# Supplementary material for: Emotional Exhaustion of Burnout Among Medical Staff and Its Association With Mindfulness and Social Support: A Single Center Study During the COVID-19 Pandemic in Japan
Source: Front Psychiatry. 2022 Mar 15;13:774919. doi: 10.3389/fpsyt.2022.774919 (PMC8965002; doi:10.3389/fpsyt.2022.774919)
Supplement: Supplementary file 1 [file Data_Sheet_1.docx]

Supplementary Materials

Title:

**Emotional exhaustion of burnout among medical staff and its association with mindfulness and social support: a single center study during the COVID-19 pandemic in Japan**

List:

Supplementary document; Distribution of measurement and internal reliability of the questionnaire

Table S1; The association between risk of emotional exhaustion and the degree of mindfulness and social support, using ordered logistic regression

Table S2; Distribution of the participants’ depression scale PHQ-9 responded

Table S3; The association between depression and the degree of mindfulness and social support, for sensitive analysis

**Supplementary document;**

**Distribution of measurement and internal reliability of the questionnaire**

The Mindful Attention Awareness Scale (MAAS) total score had high internal consistency (internal reliability of the questionnaire) with Cronbach’s α of 0.9 in our samples. Among all samples, the mean, standard deviation, median and interquartile range of the total score were mean; 68.5, the standard deviation; 12.4 and the median; 68 (IOR; 59-78), respectively. Among those with high dose exposure to SARS-CoV-2, the values were mean; 67.6, the standard deviation; 12.3 and the median; 67 (IOR; 59-77). Among those who did not have high dose exposure to SARS-CoV-2, the values were mean; 68.7, the standard deviation; 12.4 and the median; 68 (IOR; 59-78), respectively.

Similarly, the Multidimensional Scale of Perceived Social Support (MSPSS) has a high internal consistency (internal reliability of the questionnaire) for the total score with Cronbach’s α of 0.9 in our samples. Among all samples, the mean, standard deviation, median and interquartile range of the total score were mean; 39.7, the standard deviation; 7.6 and the median; 41 (IOR; 36-45), respectively. Among those with high dose exposure to SARS-CoV-2, the values were mean; 39.3, the standard deviation; 8.3 and the median; 41 (IOR; 36-45), respectively. Among those who did not have high dose exposure to SARS-CoV-2, the values were mean; 39.8, the standard deviation; 7.4 and the median; 41 (IOR; 36-45), respectively.

**S1; Table 1. The association between risk of emotional exhaustion and the degree of mindfulness and social support, using ordered logistic regression**

|  |  | Univariate regression | | Multiple regression | |
| --- | --- | --- | --- | --- | --- |
|  |  | OR | 95%CI | OR | 95%CI |
| Mindfulness | Lower | 3.85 | 2.72 - 5.44 | 3.39 | 2.36 - 4.87 |
|  | Moderate | 2.45 | 1.72 - 3.48 | 2.20 | 1.53 - 3.17 |
|  | Higher | Reference | | Reference | |
| Social support | Lower | 2.59 | 1.84- 3.65 | 1.89 | 1.31 - 2.73 |
|  | Moderate | 1.38 | 0.98 - 1.94 | 1.27 | 0.89 - 1.80 |
|  | Higher | Reference | | Reference | |
| Age (years) | 22-35 | Reference | | Reference | |
|  | 36-50 | 1.10 | 0.82 - 1.49 | 1.34 | 0.93 - 1.92 |
|  | >51 | 0.71 | 0.49 - 1.03 | 1.08 | 0.69 - 1.69 |
| Years of current work | <3 years | Reference | | Reference | |
|  | >=3 years | 1.46 | 1.11 - 1.92 | 1.29 | 0.95 - 1.76 |
| Sex | Male | Reference | | Reference | |
|  | Female | 1.09 | 0.80 - 1.48 | 0.64 | 0.44 - 0.93 |
| Job type | Doctor | Reference | | Reference | |
|  | Nurse | 2.29 | 1.60 - 3.29 | 2.37 | 1.50 - 3.74 |
|  | Other medical staff | 1.22 | 0.76 – 1.95 | 1.14 | 0.68 - 1.89 |
|  | Office worker or researcher | 1.12 | 0.76 - 1.63 | 1.35 | 0.88 - 2.09 |
| High dose exposure to SARS-CoV-2 | No | Reference | | Reference | |
|  | Yes | 1.33 | 0.96 - 1.84 | 1.24 | 0.86 - 1.78 |
| Married | No | Reference | | Reference | |
|  | Yes | 0.59 | 0.45 - 0.78 | 0.56 | 0.37 - 0.84 |
| Have children | No | Reference | | Reference | |
|  | Yes | 0.89 | 0.67 - 1.17 | 1.35 | 0.90 - 2.02 |

OR; odds ratio ,95%CI; 95% confidence interval

**S2; Table2. Distribution of the participants’ depression scale PHQ-9 responded**

|  | Frequency | Percent |
| --- | --- | --- |
| Minimal | 510 | 61.5 |
| Mild | 219 | 26.4 |
| Moderate | 70 | 8.4 |
| Moderately severe | 25 | 3.0 |
| Severe | 6 | 0.7 |
| Total | 830 | 100 |

**S3; Table3. The association between depression and the degree of mindfulness and social support, for sensitive analysis**

|  |  | Univariate logistic regression | | Multiple logistic regression | |
| --- | --- | --- | --- | --- | --- |
|  |  | OR | 95%CI | OR | 95%CI |
| Mindfulness | Lower | 9.95 | 6.54 - 15.13 | 9.27 | 5.93 - 14.47 |
|  | Moderate | 5.10 | 3.33 - 7.80 | 4.73 | 3.01 - 7.43 |
|  | Higher | Reference | | Reference | |
| Social support | Lower | 2.71 | 1.91 - 3.84 | 1.99 | 1.34 - 2.96 |
|  | Moderate | 1.31 | 0.92 - 1.87 | 1.14 | 0.77 - 1.69 |
|  | Higher | Reference | | Reference | |
| Age (years) | 22-35 | Reference | | Reference | |
|  | 36-50 | 0.84 | 0.62 - 1.14 | 1.08 | 0.73 - 1.60 |
|  | >51 | 0.56 | 0.38 - 0.82 | 0.90 | 0.50 - 1.48 |
| Years of current work | <3 years | Reference | | Reference | |
|  | >=3 years | 1.37 | 1.04 - 1.82 | 1.13 | 0.81 - 1.57 |
| Sex | Male | Reference | | Reference | |
|  | Female | 1.73 | 1.24 - 2.41 | 0.76 | 0.49 – 1.17 |
| Job type | Doctor | Reference | | Reference | |
|  | Nurse | 4.46 | 2.94 - 6.77 | 4.13 | 2.42 - 7.05 |
|  | Other medical staff | 3.02 | 1.82 - 5.01 | 2.66 | 1.50 - 4.72 |
|  | Office worker or researcher | 2.38 | 1.53 - 3.69 | 2.74 | 1.63 - 4.61 |
| High dose exposure to SARS-CoV-2 | No | Reference | | Reference | |
|  | Yes | 1.03 | 0.74 - 1.44 | 1.02 | 0.68 - 1.53 |
| Married | No | Reference | | Reference | |
|  | Yes | 0.51 | 0.39 - 0.67 | 0.63 | 0.40 - 0.98 |
| Have children | No | Reference | | Reference | |
|  | Yes | 0.66 | 0.50 - 0.88 | 0.91 | 0.58 – 1.43 |

OR; odds ratio ,95%CI; 95% confidence interval
